# Tools for Assessing the Protective Efficacy of TB Vaccines in Humans: *in vitro* Mycobacterial Growth Inhibition Predicts Outcome of *in vivo* Mycobacterial Infection

**DOI:** 10.3389/fimmu.2019.02983

**Published:** 2020-01-10

**Authors:** Rachel Tanner, Iman Satti, Stephanie A. Harris, Matthew K. O'Shea, Deniz Cizmeci, Daniel O'Connor, Agnieszka Chomka, Magali Matsumiya, Rachel Wittenberg, Angela M. Minassian, Joel Meyer, Helen A. Fletcher, Helen McShane

**Affiliations:** ^1^The Jenner Institute, Nuffield Department of Medicine, University of Oxford, Oxford, United Kingdom; ^2^Institute of Microbiology and Infection, College of Medical and Dental Sciences, The University of Birmingham, Birmingham, United Kingdom; ^3^Department of Paediatrics, University of Oxford, Oxford, United Kingdom; ^4^London School of Hygiene and Tropical Medicine, London, United Kingdom

**Keywords:** Tuberculosis, vaccine, BCG, mycobacterial growth inhibition assay, MGIA

## Abstract

Tuberculosis (TB) remains a leading global cause of morbidity and mortality and an effective new vaccine is urgently needed. A major barrier to the rational development of novel TB vaccines is the lack of a validated immune correlate or biomarker of protection. Mycobacterial Growth Inhibition Assays (MGIAs) provide an unbiased measure of ability to control mycobacterial growth *in vitro*, and may represent a functional correlate of protection. However, the biological relevance of any potential correlate can only be assessed by determining the association with *in vivo* protection from either a controlled mycobacterial infection or natural development of TB disease. Our data demonstrate that the direct MGIA using peripheral blood mononuclear cells (PBMC) is measuring a biologically relevant response that correlates with protection from *in vivo* human BCG infection across two independent cohorts. This is the first report of an MGIA correlating with *in vivo* protection in the species-of-interest, humans, and furthermore on a per-individual as well as per-group basis. Control of mycobacterial growth in the MGIA is associated with a range of immune parameters measured post-BCG infection *in vivo* including the IFN-γ ELISpot response, frequency of PPD-specific IFN-γ or TNF-α producing CD4+ T cells and frequency of specific sub-populations of polyfunctional CD4+ T cells. Distinct transcriptomic profiles are associated with good vs. poor mycobacterial control in the MGIA, with good controllers showing enrichment for gene sets associated with antigen processing/presentation and the IL-23 pathway, and poor controllers showing enrichment for hypoxia-related pathways. This study represents an important step toward biologically validating the direct PBMC MGIA for use in TB vaccine development and furthermore demonstrates the utility of this assay in determining relevant immune mechanisms and pathways of protection.

## Introduction

Tuberculosis (TB) is the leading cause of mortality worldwide from a single infectious agent, with an estimated 10 million new cases and 1.5 million deaths in 2018 (1). The high prevalence of latent *Mycobacterium tuberculosis* (*M.tb*) infection, convergence of the TB and HIV epidemics, emergence of multi-drug resistant strains of *M.tb* and the logistics of delivering complex treatment regimens in endemic countries mean that vaccination is widely recognized as a critical component of the strategy to control TB (2). BCG is the only currently available TB vaccine and is widely used in many parts of the world to protect against severe forms of TB in infancy (3). However, BCG-induced protection against pulmonary disease in adults (the most common form of TB) is extremely variable (4), and there is an urgent need for a new, more effective vaccine.

A major barrier to the rational development of novel TB vaccines is the lack of a validated immune correlate or biomarker of protection. A correlate of protection may be defined as an immune marker statistically correlated with vaccine efficacy (equivalently predictive of vaccine efficacy) that may or may not be a mechanistic causal agent of protection (5). Identification of such a correlate would expedite TB vaccine development, allowing the down-selection of candidates at an early stage of development and providing a relevant measure of immunogenicity in phase I clinical trials. The TB vaccine field has to date focussed largely on identifying T cell signatures of efficacy, but correlates of protection studies and the outcome of the MVA85A efficacy trial have cast doubt on the sufficiency of the cell-mediated response alone to confer protection by vaccination (6, 7). Mycobacterial growth inhibition assays (MGIAs) are functional assays that offer an alternative to measuring predefined individual immune parameters (8). Rather, they aim to provide an unbiased measure of the ability of whole blood or cell samples to control mycobacterial growth *in vitro*, and as such may represent a correlate of protection without the need to understand the underlying immune mechanisms.

We have recently worked toward optimizing, standardizing, and harmonizing the direct MGIA using peripheral blood mononuclear cells (PBMC) for the evaluation of prophylactic TB vaccine candidates (9, 10). This assay is adapted from the methods of Wallis et al. (11) and aims to simplify the MGIA to maximize reproducibility, applying standardized reagents and equipment to aid in transferability. The direct PBMC MGIA has yielded promising results, demonstrating an ability to detect BCG-induced responses across different laboratories (10, 12–14). In the absence of an effective vaccine, identifying a response to BCG vaccination in populations where it is known to have high efficacy [such as the UK (4)] is currently a ‘gold standard' for assessing new models for TB vaccine evaluation. However, even within such populations BCG-induced protection is variable and gross comparison by group central tendencies can be a crude measure when it is unclear whether such groups truly differ in terms of *in vivo* protection. The biological relevance of a potential correlate such as the MGIA can only be confirmed by demonstrating an association with *in vivo* protection from either a controlled human mycobacterial infection or natural development of TB disease. Similar assessments are being conducted of the malaria growth inhibition assay (GIA) in relation to protection from controlled human malaria infection (CHMI) and immunoepidemiology studies (15).

While deliberate infection models are in use for other infectious pathogens (16–19), it is not ethically viable to infect humans with virulent *M.tb*. We have previously developed and described an *in vivo* human challenge model using intradermal *Mycobacterium bovis* BCG vaccination as a surrogate (20–22). The rationale is that an effective vaccine against *M.tb* should also reduce the replication of BCG. BCG represents a feasible challenge agent, as it causes self-contained limited infection in immunocompetent humans and is licensed for human use. Volunteers are infected with intradermal BCG and 2 weeks later, BCG is quantified from skin biopsies of the challenge site. This model has demonstrated ability to detect differences in protective immunity induced by BCG vaccination in UK adults (20, 21). The aim of the study presented was to use samples from these human BCG infection studies to provide biological validation of the direct PBMC MGIA. We compared *in vitro* mycobacterial growth in the MGIA with *in vivo* growth in skin biopsies from the same BCG infected human volunteers across different cohorts, and explored some of the underlying cellular and humoral immune mechanisms that may be associated with vaccine-induced control of mycobacterial growth.

## Methods

### Human Studies

Samples were taken from two studies of BCG infection in healthy UK volunteers aged 18–50 years, for whom latent *M.tb* infection was excluded by negative *ex vivo* interferon-gamma ELISpot responses to ESAT-6 and CFP-10 peptides. All volunteers gave written informed consent before participation and the trials were carried out in accordance with the ethical principles set forth in the Declaration of Helsinki as agreed by the World Medical Association General Assembly (Washington, 2002), ICH Good Clinical Practice (GCP) and local regulatory requirements.

Study 1 was approved by the Oxfordshire Research Ethics Committee A (REC reference 07/Q1604/3) and the full details including inclusion and exclusion criteria have been described elsewhere (21). Participants were either BCG-naïve (*n* = 12) or historically BCG-vaccinated (*n* = 12); historically BCG-vaccinated volunteers were immunized between 8 months and 10 years prior to enrolment with a median time since vaccination of 4 years. A sufficient quantity of cells and autologous serum was available for MGIA studies from *n* = 11 volunteers from each group. All volunteers received intradermal challenge with BCG (SSI; 0.05 mL; diluted in saline to 0.1 mL) from a vial containing 2–8 × 10^6^ colony-forming units (CFU)/mL, giving a final dose of ~1–4 × 10^5^ CFU into the upper arm (deltoid insertion).

Study 2 (NCT01194180) was approved by the Medicines and Healthcare Products Regulatory Agency (EudraCT 2010-018425-19) and the Berkshire Research Ethics Committee (REC reference 10/H0505/31). Full details are provided elsewhere (20). Participants were assigned to group A (BCG-naive; no vaccine received; *n* = 11), group B (BCG-naive at baseline; received intradermal MVA85A, dose 1 × 10^8^ plaque-forming units pfu; *n* = 12), group C (BCG-vaccinated at baseline between 8 and 38 years prior to enrolment; median time since vaccination 10 years; *n* = 13), or group D (BCG-vaccinated at baseline between 6 and 33 years prior to enrolment; median time since vaccination 10.5 years; received intradermal MVA85A, dose 1 × 10^8^ pfu; *n* = 12) based on their prior BCG vaccination status and meeting inclusion criteria. A sufficient quantity of cells and autologous plasma was available for MGIA studies from *n* = 8 volunteers from group A, *n* = 11 volunteers from group B, *n* = 12 volunteers from group C, and *n* = 12 volunteers from group D. All participants were challenged with a standard vaccine dose of intradermal BCG (SSI; 0.1 mL containing 2–8 × 10^5^ CFU). Those in groups B and D were challenged 4 weeks after MVA85A vaccination.

For both studies, a punch biopsy was performed at the challenge site 2 weeks after BCG infection. Freezing, homogenization and culturing of biopsies as well as DNA extraction and qPCR methods have been previously described (20).

### Sample Processing

PBMC were isolated from heparinized peripheral blood samples using density centrifugation. Cells were counted, resuspended in fetal bovine serum (FBS) and incubated at 4°C for 20 min. An equal volume of FBS containing 20% dimethylsulfoxide (DMSO) was then added and cells aliquoted at a concentration of 5–10 × 10^6^ cells per cryovial, frozen overnight at −80°C and transferred to LN_2_ the following day. Cryopreserved PBMC were thawed in a water bath at 37°C until a small amount of frozen material remained. Samples were gradually added to 10 ml RPMI (containing 10% FBS and 2 mM l-glutamine) and centrifuged at 1,500 rpm for 7 min. Supernatants were poured off and cells resuspended at an approximate concentration of 2–3 × 10^6^ cells per ml of RPMI (containing 10% fetal calf serum and 2 mM l-glutamine), and 2 μl/ml of 25 U benzonase added to each tube. Cells were rested at 37°C for 2 h with 5% CO_2_ before counting using a CASY Counter (Roche).

### Direct PBMC MGIA

3 × 10^6^ PBMC and ~500 CFU BCG Pasteur in a total volume of 480 μl RPMI (containing 2 mM l-glutamine and 25 mM HEPES), plus 120 μl autologous serum (Study 1) or plasma (Study 2) (matched to volunteer and time-point) per well were added to a 48-well-plate (total volume 600 μl per well). Co-cultures were incubated at 37°C for 96 h and then added to 2 ml screw-cap tubes and centrifuged at 12,000 rpm for 10 min. During this time, 500 μl sterile water was added to each well to lyse adherent monocytes. Supernatants were removed from the 2 ml screw-cap tubes, and water from the corresponding well added to the pellet. Tubes were pulse vortexed and the lysate transferred to BACTEC MGIT tubes supplemented with PANTA antibiotics and OADC enrichment broth (Becton Dickinson, UK). Tubes were placed on the BACTEC 960 machine (Becton Dickinson, UK) and incubated at 37°C until the detection of positivity by fluorescence. On day 0, duplicate direct-to-MGIT viability control tubes were set up by inoculating supplemented BACTEC MGIT tubes with the same volume and CFU of mycobacteria as the samples. The time to positivity (TTP) read-out was converted to log_10_ CFU using stock standard curves of TTP against inoculum volume and CFU. Results are presented as growth ratio (log_10_ CFU of sample/log_10_ CFU of growth control) to correct for any batch-to-batch variability in inoculum. For the purposes of the gene expression analysis, BCG-vaccinated volunteers were classified as “good” or “poor” controllers defined as having MGIA growth ratio values below or above the group median, respectively.

### Intracellular Cytokine Staining (ICS)

Whole blood was incubated with 1 μg/mL αCD28, 1 μg/mL αCD49d (BD Biosciences) and stimulated with 20 μg/ml PPD (SSI, Denmark), 5 μg/ml staphylococcal enterotoxin B (Sigma Aldrich), or no antigen (unstimulated). Samples were incubated at 37°C in 5% CO_2_ for 6 h, 3 μg/ml Brefeldin-A (Sigma Aldrich) was added, and samples were incubated for another 6 h in a 37°C water bath. Samples were then treated with 2 mM ethylenediaminetetraacetic acid (Gibco), and red blood cells were lysed using FACS Lysing solution (BD Biosciences) and samples were frozen in PBS with 10% DMSO for batched ICS staining. Frozen samples were thawed, permeabilised and incubated with antibodies against CD3 (AF700), IFN-γ (PE-Cy7 (eBioscience); CD4 (APC), CD14 (Pacific blue), TNF-α (PerCP-Cy5.5), and IL-17 (AF488) (BioLegend); CD8 (APC-H7) (Becton Dickinson) and IL-2 (PE) (Beckman Coulter). Samples were acquired on an LSR II flow cytometer (BD Biosciences) and responses analyzed using FlowJo version 8.8.7 (Tree Star Inc., Ashland, USA). Cytokines were measured as the frequency of singlet CD14– CD3+ T cells, CD4+ T cells, and CD8+ T cells. Data are presented as percentages of cytokine-positive cells minus responses in unstimulated cells; the gating strategy is shown in Supplementary Figure 1.

### *Ex vivo* Interferon-Gamma ELISpot

ELISpots were performed using a human IFN-γ ELISpot kit (capture mAb D1K, Mabtech). Duplicate wells containing 3 × 10^5^ PBMC were stimulated for 18 h with purified protein derivative (PPD) from *M.tb* SSI at a concentration of 20 μg/ml. Staphylococcal enterotoxin B (Sigma) was used as a positive control at a concentration of 10 μg/ml, media alone was used as a negative control and unstimulated PBMCs were used to measure background IFN-γ production. Results are reported as spot forming cells (SFC) per million PBMC, calculated by subtracting the mean count of the unstimulated wells from the mean count of duplicate PPD-stimulated wells and correcting for the number of PBMC in the well.

### Enzyme-Linked Immunosorbent Assay (ELISA)

Ninety-six well flat-bottom microtiter plates were coated overnight with 50 μl per well of BCG SSI (1 × 10^4^ CFU/well). Samples from Study 2 were prepared by diluting test serum and positive/negative control serum 1:10 in casein blocking buffer. The plates were washed 6 times with PBS 0.05% Tween 20, followed by blocking with 200 μl casein per well for 1 h at room temperature. The blocking solution was removed, 50 μl of sample was added per well (50 μl casein to the negative control wells), and the plates incubated for 2 h at room temperature. The plates were then washed 6 times with PBS 0.05% Tween 20. Secondary antibody (goat anti-human γ-chain whole IgG alkaline phosphatase conjugate) was diluted 1:1,000 in casein, vortexed and 50 μl added per well. The plates were then incubated for 1 h at room temperature and washed 6 times with PBS Tween 20. One hundred microliter of p-nitrophenyl phosphate (pNPP) development buffer was added to each well and the plates incubated at room temperature in the dark for 30 min. OD_405_ was read using a Model 550 Microplate Reader (Bio-Rad, UK).

### Gene Expression Microarray Analysis

Gene expression microarrays were performed as previously described (23). Briefly, 2 × 10^6^ cryopreserved PBMC were stimulated for 12 h with either R10 medium alone or R10 medium containing 1 × 10^6^ CFU of BCG (SSI). Supernatants were then removed and the PBMC resuspended in 350 μl RLT buffer (Qiagen) containing 10 μl/ml β-mercaptoethanol and frozen at −20°C. RNA was extracted using an RNeasy kit (Qiagen) according to the manufacturer's instructions. RNA quantity and quality was assessed using a Nanodrop ND-1000 Spectrophotometer and an Agilent Bioanalyzer (Agilent RNA 6000 Nano Kit). Seven hundred fifty nanogram of amplified complementary RNA was labeled and hybridized to Illumina Human HT-12 v4 beadchips according to the manufacturer's instructions. Beadchips were scanned on an Illumina iScan machine, and data extracted using the GenomeStudio software.

Transcriptomic analysis was performed in R version 3.5.2 (2018-12-20). Microarray data (GSE58636) were analyzed for differential expression using R package limma version 3.38.3. To account for between-patient correlations, limma *duplicateCorrelation* function was used. A linear model was fit using limma *lmFit* function focusing on samples collected at 2 weeks post-infection from group C. Differential expression between good and poor controllers in the MGIA was evaluated using moderated *t*-statistics and the *p*-values were adjusted using Benjamini and Hochberg's (BH) method. Genes with adjusted *p* < 0.01 and log_2_ fold change > 0.5 were identified as significantly differentially expressed. Genes with low average expression (normalized intensity < 5) were excluded. The gene list was collapsed to unique gene identifiers by removing duplicate probes mapping to gene identifiers when the gene list was ordered by limma *p*-value. Gene set enrichment analysis (GSEA) was performed on the entire list of filtered genes, ranked by their t-statistic from limma, using “GseaPreranked” tool in the Java based desktop application of GSEA version 4.0.0 (24). Analysis was completed using the gene sets in the “C2: curated gene sets” database curated from biomedical literature and domain experts (http://www.broadinstitute.org/gsea/msigdb/index.jsp). Gene sets with fewer than 15 genes or more than 500 genes were excluded. GSEA was run using remaining gene sets (*n* = 3,136) with 1,000 permutations of the gene list.

### Statistical Analysis

Statistical analysis of the cellular data was performed using GraphPad Prism version 7.05 and IBM SPSS version 25. Normality of data was determined using a Shapiro-Wilk test. For parametric data with multiple groups, a one-way ANOVA or repeated-measured ANOVA was conducted followed by a Dunn's post-test (comparison of all groups) or Sidak's multiple comparisons test (comparison of preselected pairs of groups). For comparisons between two groups of normally-distributed data, a *t*-test or paired *t*-test was used. For non-parametric data with multiple groups, a Kruskal-Wallis or Friedman test was conducted followed by a Dunn's post-test. For comparisons between two groups of non-parametric data or small sample size, a Mann Whitney or Wilcoxon matched-pairs signed rank test was conducted. A Spearman's rank correlation was used to determine associations between two different measures. Differences were considered statistically significant at *p* < 0.05.

## Results

### Mycobacterial Growth in the MGIA Is Inhibited in BCG-Vaccinated Compared With Naïve Volunteers and Correlates With BCG Recovered From Biopsy Following *in vivo* BCG Infection

**Figure 1:**
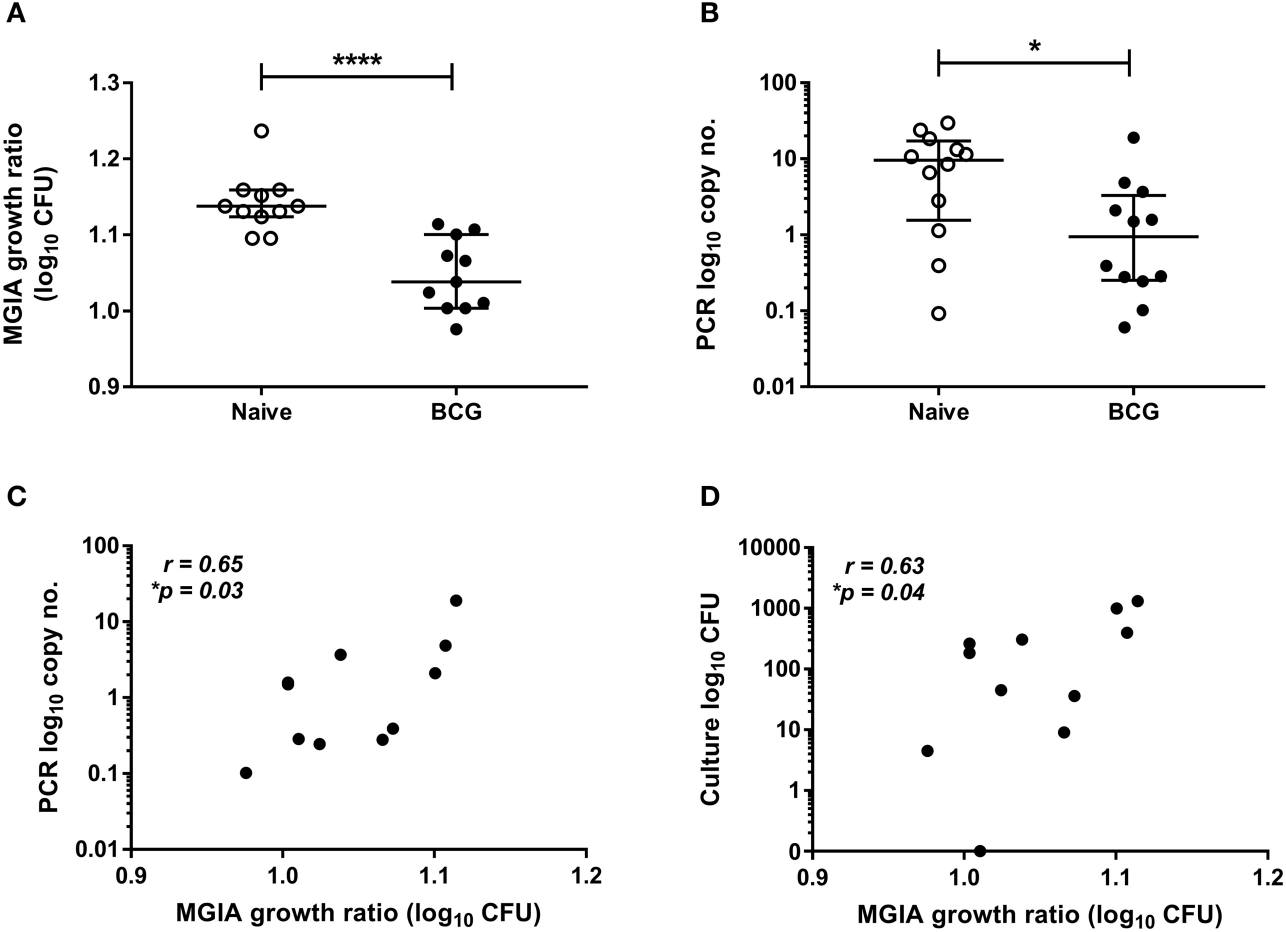
Mycobacterial growth in the MGIA is inhibited in BCG-vaccinated compared with naïve volunteers and correlates with BCG recovered from biopsy following *in vivo* BCG infection (Study 1). Samples were taken from Study 1. *n* = 24 healthy human volunteers, half of whom were BCG-naïve and half of whom were historically BCG-vaccinated, were infected with intradermal BCG. The direct PBMC MGIA was conducted on cells and serum taken at the day of infection and the ratio of mycobacterial growth at the end of the 96 h culture relative to an inoculum control was determined **(A)**. As previously reported, the BCG load was quantified from skin biopsy specimens at the site of challenge 2 weeks later using PCR **(B)**. The association between mycobacterial growth in the MGIA and BCG recovered from biopsies in the BCG vaccinated group by both PCR **(C)** and culture CFU **(D)** was determined. Bars represent the median values with interquartile range (IQR). For **(A,B)** a Mann-Whitney U test was performed, where *p < 0.05 and ****p < 0.0001. For **(C,D)** a Spearman's correlation was performed. MGIA growth ratio = log_10_(CFU of sample/CFU of control).

Samples were taken from a previously-described study of 24 healthy human volunteers (Study 1), half of whom were BCG-naïve and half of whom were historically BCG-vaccinated. All volunteers were challenged with intradermal BCG and BCG load quantified from skin biopsy specimens at the site of infection 2 weeks later using qPCR and culture CFU (21). The direct PBMC MGIA was conducted using cells and serum taken on the day of infection. There was significantly improved control of mycobacterial growth in the historically BCG-vaccinated volunteers compared with the naïve group (Figure 1A, *p* < 0.0001, Mann Whitney test). This is consistent with the *in vivo* result previously reported, where there was significantly lower BCG recovery from biopsies of historically BCG-vaccinated compared with BCG-naïve volunteers using qPCR (Figure 1B, *p* = 0.02, Mann Whitney test) but not culture CFU, although the two measures did correlate (21). There was a significant correlation between growth in the MGIA and BCG recovered from biopsies in the BCG-vaccinated group by both qPCR (Figure 1C, *r* = 0.65, *p* = 0.03, Spearman's correlation) and culture CFU (Figure 1D, *r* = 0.63, *p* = 0.04, Spearman's correlation), but no associations were observed in the naïve group (data not shown).

**Figure 2:**
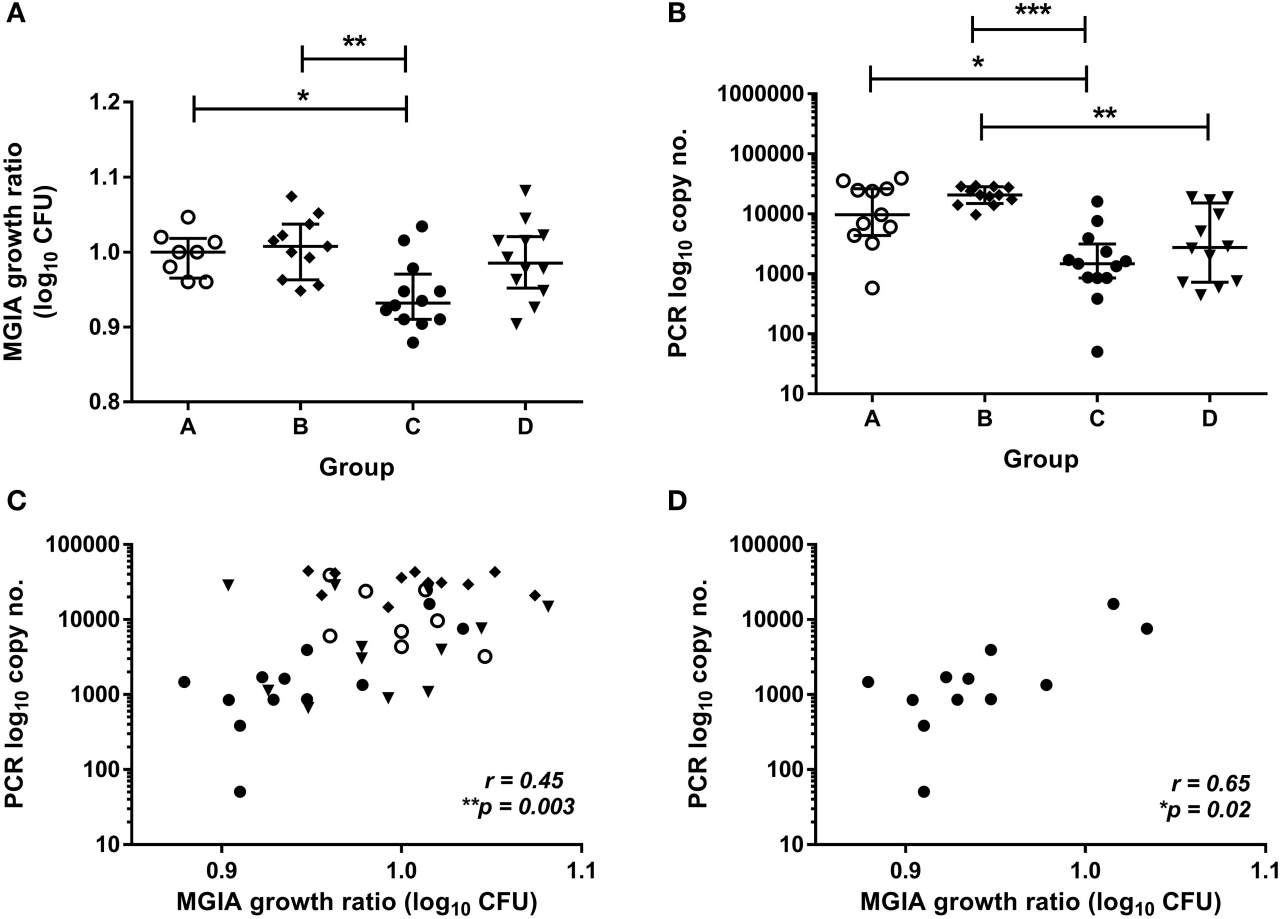
Mycobacterial growth in the MGIA is inhibited in BCG-vaccinated compared with naïve volunteers and correlates with BCG recovered from biopsy following *in vivo* BCG infection (Study 2). Samples were taken from Study 2. *n* = 48 healthy human volunteers were assigned to groups A and B (BCG-naïve) or groups C and D (historically BCG vaccinated). Groups B and D received the candidate TB vaccine MVA85A. All volunteers were then infected with intradermal BCG. The direct PBMC MGIA was conducted on cells and plasma taken at the day of infection and the ratio of mycobacterial growth at the end of the 96 h culture relative to an inoculum control was determined **(A)**. As previously reported, the BCG load was quantified from skin biopsy specimens at the site of challenge 2 weeks later using PCR **(B)**. The association between mycobacterial growth in the MGIA and BCG recovered from biopsies was determined for all groups combined **(C)** and for the BCG-vaccinated group (group C) only **(D)**. Bars represent the median values with IQR. For **(A)** a Kruskal Wallis test with Dunn's multiple comparisons was performed and for **(B)** a one-way ANOVA with Tukey's multiple comparisons test was performed, where *p < 0.05, **p < 0.005, and ***p < 0.0005. For **(C,D)** a Spearman's correlation was performed. MGIA growth ratio = log_10_(CFU of sample/CFU of control).

To validate this finding in a second independent cohort, samples were taken from a previously-described study of 48 healthy human volunteers (Study 2) assigned to groups A and B (BCG-naïve) or groups C and D (historically BCG-vaccinated). Groups B and D received the candidate TB vaccine MVA85A. All volunteers were infected with intradermal BCG (at 4 weeks post-MVA85A in groups B and D), and skin biopsies of the challenge site were taken 2 weeks later. BCG was quantified by qPCR and culture CFU (20). The direct PBMC MGIA was conducted using cells and serum taken from all groups at the day of infection. There was significantly improved control of mycobacterial growth in the historically BCG-vaccinated group compared with the naïve group or the MVA85A alone group (group A vs. C and B vs. C) (Figure 2A, *p* = 0.04 and *p* = 0.0063, respectively, one-way ANOVA with Tukey's multiple comparisons test). This is consistent with the *in vivo* result previously reported, where volunteers with a history of BCG showed some degree of protective immunity to intradermal BCG infection using qPCR (but not culture CFU) (20). BCG-vaccinated volunteers had significantly lower BCG recovery from biopsy than naïve individuals or those who received MVA85A only (group A vs. C and B vs. C) (Figure 2B, *p* = 0.02 and *p* = 0.0001, respectively, Kruskal-Wallis test with Dunn's multiple comparisons). Individuals who received BCG followed by MVA85A had significantly lower BCG recovery from biopsy than those who received MVA85A only (group B vs. D) (Figure 2A, *p* = 0.0062, Kruskal-Wallis test with Dunn's multiple comparisons). There was a significant correlation between growth in the MGIA and BCG recovered from biopsies by qPCR when all groups were combined (Figure 2C, *r* = 0.45, *p* = 0.003, Spearman's correlation) and in the historically BCG-vaccinated group (group C) alone (Figure 2D, *r* = 0.65, *p* = 0.02, Spearman's correlation), but not in groups A, B, or D alone (data not shown). Group C was our group of interest as these volunteers showed a signal of protection following both *in vivo* and *in vitro* BCG infection, and BCG is known to be efficacious in the UK population under study (4).

### Frequency of Sub-populations of Cytokine-Producing CD4+ T Cells Is Associated With Improved Control of Mycobacterial Growth in the MGIA

**Figure 3:**
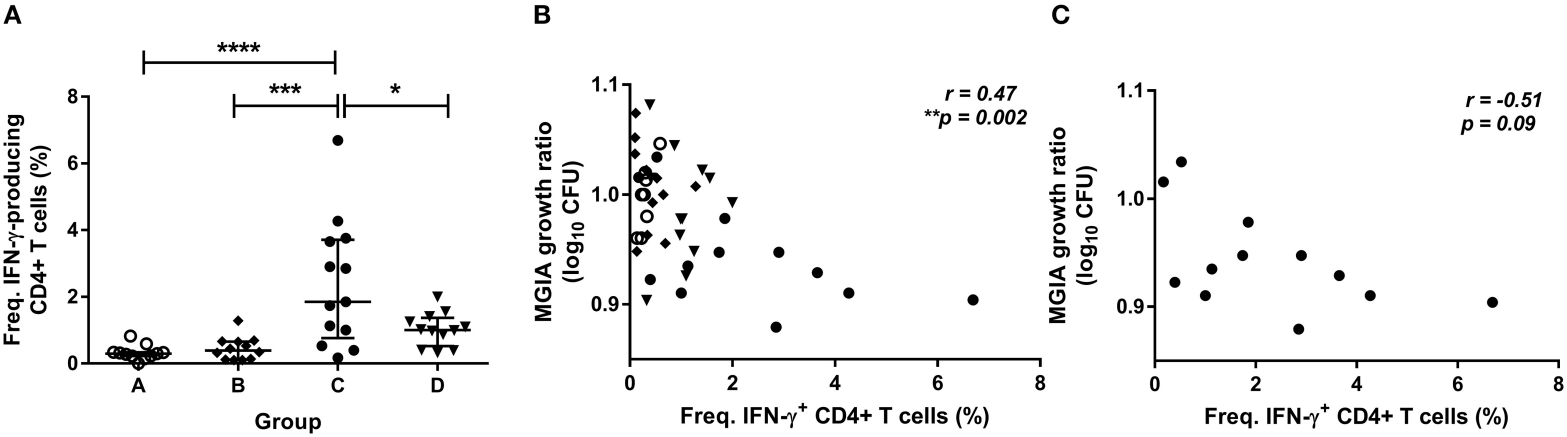
Frequency of cytokine-producing specific CD4+ T cells is associated with improved control of mycobacterial growth in the MGIA. Samples were taken from Study 2. *n* = 48 healthy human volunteers were assigned to groups A and B (BCG-naïve) or groups C and D (historically BCG vaccinated). Groups B and D received the candidate TB vaccine MVA85A. All volunteers were then infected with intradermal BCG, and frequency of PPD-specific IFN-γ producing CD4+ T cells were measured at 2 weeks post-infection using whole blood ICS **(A)**. The direct PBMC MGIA was conducted on cells and serum taken at the day of challenge and the ratio of mycobacterial growth at the end of the 96 h culture relative to an inoculum control was determined. The association between mycobacterial growth in the MGIA and the frequency of PPD-specific IFN-γ producing CD4+ T cells was determined for all groups combined **(B)** and for the BCG-vaccinated group (group C) only **(C)**. Bars represent the median values with IQR. For **(A)**, a one-way ANOVA with Tukey's multiple comparisons was performed where *p < 0.05, ***p < 0.0005, and ****p < 0.0001. For **(B,C)** a Spearman's correlation was performed. MGIA growth ratio = log_10_(CFU of sample/CFU of control).

To determine how immune responses stimulated by *in vivo* BCG infection relate to control of mycobacterial growth following *in vitro* infection, we analyzed a variety of parameters at 2 weeks post-infection in samples from Study 2. Using whole blood ICS, significantly more PPD-specific CD4+ T cells producing IFN-γ were measured in the BCG-vaccinated volunteers (group C) compared with naïve (group A), MVA85A-vaccinated (group B) and BCG-vaccinated volunteers boosted with MVA85A (group D) at 2 weeks post-BCG infection (Figure 3A, *p* < 0.0001, *p* = 0.0002, *p* = 0.01, respectively, one-way ANOVA with Tukey's multiple comparisons test). There was a significant inverse correlation between mycobacterial growth in the MGIA and PPD-specific CD4+ T cells producing IFN-γ when all groups were combined (Figure 3B, *r* = −0.47, *p* = 0.002, Spearman's correlation) and a trend in the historically BCG-vaccinated group alone (group C), although this was not statistically significant (Figure 3C, *r* = −0.51, *p* = 0.09). Perhaps unsurprisingly, as the majority of PPD-specific CD4+ T cells produce both IFN-γ and TNF-α simultaneously, similar patterns were observed for TNF-α with significantly more PPD-specific CD4+ T cells producing TNF-α measured in the BCG-vaccinated volunteers (group C) compared with naïve (group A), MVA85A-vaccinated (group B), and BCG-vaccinated volunteers boosted with MVA85A (group D) (*p* < 0.0001, *p* = 0.0001, *p* = 0.01, respectively, one-way ANOVA with Tukey's multiple comparisons test; data not shown). There was a significant inverse correlation between mycobacterial growth in the MGIA and PPD-specific CD4+ T cells producing TNF-α when all groups were combined (*r* = −0.48, *p* = 0.001, Spearman's correlation; data not shown) and a trend in the historically BCG-vaccinated group alone (group C), although this was not statistically significant (*r* = −0.51, *p* = 0.09; data not shown). There was a similar pattern between groups for PPD-specific CD8+ T cells producing IFN-γ, and a significant inverse correlation with the MGIA when all groups were combined and a trend in group C alone (*r* = −0.54, *p* = 0.0002, and *r* = −0.56, *p* = 0.05, respectively; data not shown). No such associations were observed in groups A, B, or D when taken alone (data not shown).

In support of these findings, significantly higher levels of PPD-specific IFN-γ were detected in BCG-vaccinated volunteers (group C) compared with naïve (group A) or MVA85A-vaccinated volunteers (group B) using IFN-γ ELISpot (Supplementary Figure 2A, *p* < 0.0001 and *p* = 0.0004, respectively, one-way ANOVA with Tukey's multiple comparisons test). Responses were also significantly higher in individuals who received BCG alone compared with those who received BCG followed by MVA85A (Supplementary Figure 2A, *p* = 0.006, one-way ANOVA with Tukey's multiple comparisons test). There was a significant inverse correlation between mycobacterial growth in the MGIA and PPD-specific ELISpot response when all groups were combined (Supplementary Figure 2B, *r* = −0.37, *p* = 0.02, Spearman's correlation) and a trend in the historically BCG-vaccinated group alone (group C), although this was not statistically significant (Supplementary Figure 2C, *r* = −0.49, *p* = 0.11). No associations were observed in groups A, B, or D when taken alone (data not shown).

**Figure 4:**
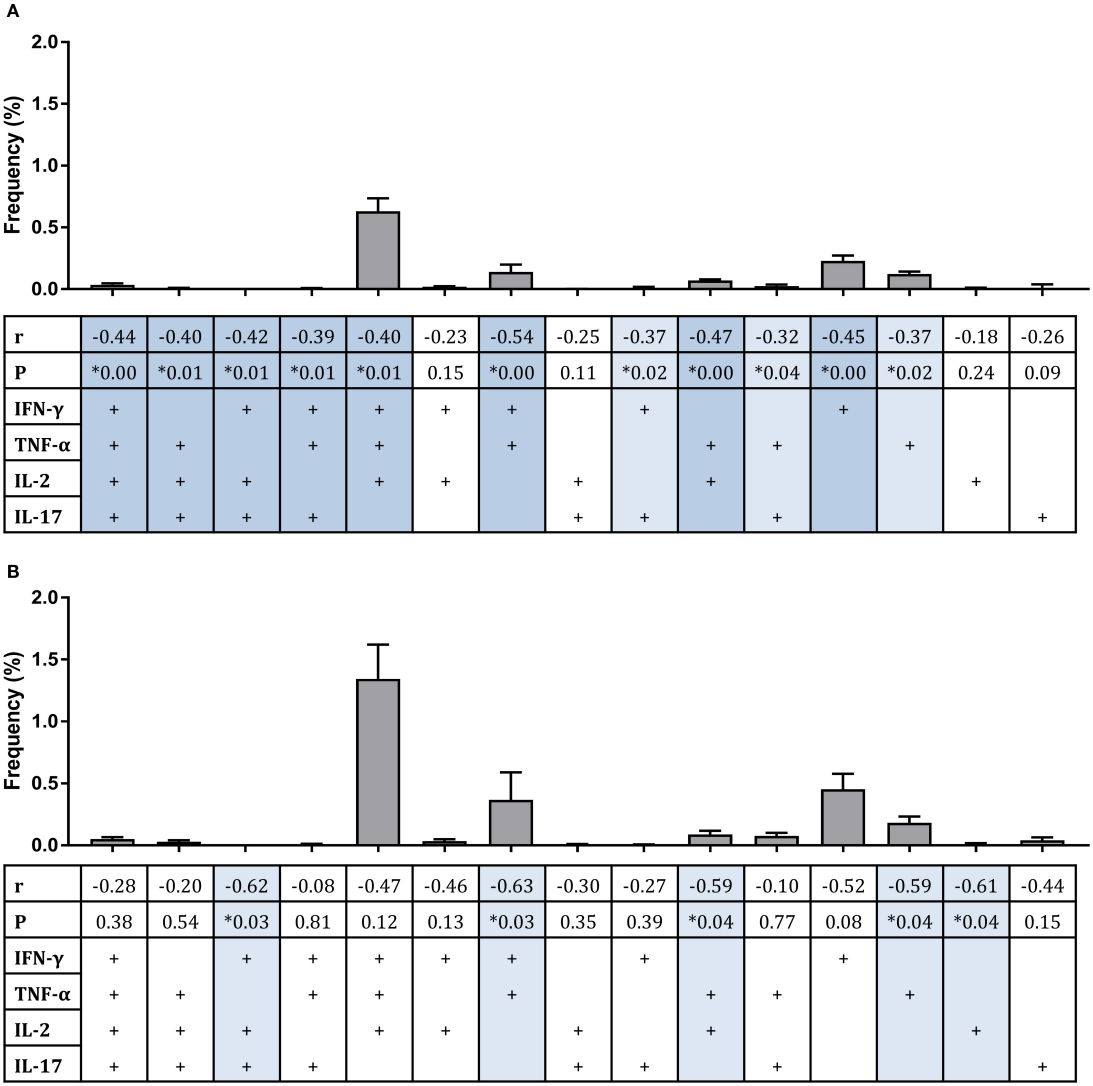
Frequency of sub-populations of cytokine-producing specific CD4+ T cells is associated with improved control of mycobacterial growth in the MGIA. Samples were taken from Study 2. *n* = 48 healthy human volunteers were assigned to groups A and B (BCG-naïve) or groups C and D (historically BCG vaccinated). Groups B and D received the candidate TB vaccine MVA85A. All volunteers were then infected with intradermal BCG, and the frequency of PPD-specific CD4+ T cells producing each of the possible expression profile permutations of the cytokines IFN-γ, TNF-α, IL-2, and IL-17 was determined by flow cytometry using whole blood samples taken at 2 weeks post-infection. The direct PBMC MGIA was conducted on cells and plasma taken at the day of challenge and the ratio of mycobacterial growth at the end of the 96 h culture relative to an inoculum control was determined. The association between mycobacterial growth in the MGIA at day of challenge and frequency of CD4+ T cells producing each profile was determined for all groups combined **(A)** or for the BCG-vaccinated group (group C) only **(B)** using Spearman's rank correlation, where light blue shading indicates a *p* < 0.05 and darker blue shading indicates a *p* < 0.01. Gray bars indicate the mean frequency of each sub-population of cells with the SEM.

Considering polyfunctional CD4+ T cells, the dominant responder population at 2 weeks post-infection for all groups combined and for historically BCG-vaccinated individuals alone (group C) was IFN-γ, TNF-α, and IL-2 triple-cytokine producing cells; while the second most abundant population was single IFN-γ producers (Figures 4A,B). There were significant inverse correlations between frequencies of several subsets and mycobacterial growth in the MGIA when all groups were combined (Figure 4A). Subsets of cells that correlated significantly included IFN-γ, TNF-α, IL-2, and IL-17 quadruple-cytokine producing cells (Figure 4A, *r* = −0.44, *p* = 0.003, Spearman's correlation) and all permutations of triple-cytokine producing cells. The strongest association was with IFN-γ and TNF-α double-cytokine producing cells (Figure 4A, *r* = −0.54, *p* = 0.0002, Spearman's correlation), which also represented one of the largest cell populations. This correlation was also observed in BCG-vaccinated volunteers alone (group C) (Figure 4B, *r* = −0.63, *p* = 0.03, Spearman's correlation). Other associations that were replicated in this group were with IFN-γ, IL-2, IL-17 triple-positive cells (Figure 4B, *r* = −0.62, *p* = 0.03, Spearman's correlation), TNF-α and IL-2 double-positive cells (Figure 4B, *r* = −0.59, *p* = 0.04, Spearman's correlation), and TNF-α single-positive cells (Figure 4B, *r* = −0.59, *p* = 0.04, Spearman's correlation).

### Specific Antibody Responses Are Induced by BCG Vaccination and IgG Responses Are Weakly Associated With Control of Mycobacterial Growth in the MGIA

**Figure 5:**
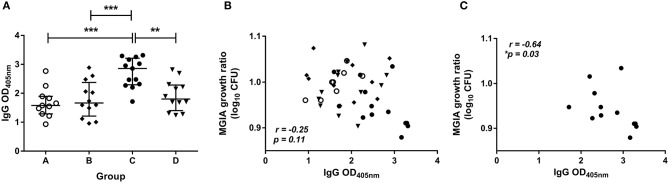
Specific IgG ELISA responses are induced by BCG vaccination and are associated with improved control of mycobacterial growth in the MGIA. Samples were taken from Study 2. *n* = 48 healthy human volunteers were assigned to groups A and B (BCG-naïve) or groups C and D (historically BCG vaccinated). Groups B and D received the candidate TB vaccine MVA85A. All volunteers were then infected with intradermal BCG, and BCG-specific IgG responses **(A)** were measured at 2 weeks post-infection. The direct PBMC MGIA was conducted on cells and plasma taken at the day of challenge and the ratio of mycobacterial growth at the end of the 96 h culture relative to an inoculum control was determined. The association between mycobacterial growth in the MGIA and the BCG-specific IgG ELISA response was determined for all groups combined **(B)** and for the BCG-vaccinated group (group C) only **(C)**. Bars represent the median values with IQR. For **(A)** a one-way ANOVA with Tukey's multiple comparisons was performed where **p < 0.005 and ***p < 0.0005. For **(B,C)** a Spearman's correlation was performed. MGIA growth ratio = log_10_(CFU of sample/CFU of control).

The level of BCG-specific IgG, IgA, and IgM in the plasma of volunteers from Study 2 was measured by ELISA at 2 weeks post-BCG infection. There were significantly higher levels of specific IgG in the plasma from BCG-vaccinated volunteers (group C) compared with naïve (group A), MVA85A-vaccinated (group B), or BCG-vaccinated volunteers boosted with MVA85A (group D) (Figure 5A, *p* = 0.0002, *p* = 0.0007, *p* = 0.004, respectively, one-way ANOVA with Tukey's multiple comparisons test). There were also significantly higher levels of specific IgA in the plasma from BCG-vaccinated volunteers compared with who received MVA85A alone (*p* = 0.02, Kruskal Wallis test with Dunn's multiple comparisons, data not shown). Levels of IgM were low and there were no significant differences between groups (data not shown). There was a trend toward an inverse correlation between mycobacterial growth in the MGIA and level of plasma IgG when all groups were combined (Figure 5B, *r* = −0.25, *p* = 0.11, Spearman's correlation), and a statistically significant inverse correlation between these measures in the BCG-vaccinated group (group C) alone (Figure 5C, *r* = −0.64, *p* = 0.03, Spearman's correlation), but not in groups A, B, or D when taken alone (data not shown). There was a trend toward an inverse correlation between BCG recovered from biopsies by qPCR and level of plasma IgG when all groups were combined (*r* = −0.26, *p* = 0.07, Spearman's correlation) and in the historically BCG-vaccinated group (group C) alone (*r* = −0.55, *p* = 0.07, Spearman's correlation) but this was not statistically significant (data not shown). There was no correlation between MGIA response and levels of IgA or IgM. There was a significant inverse correlation between BCG recovered from biopsies by qPCR and levels of IgA when all groups were combined (*r* = −0.58, *p* = 0.02) and but not in the historically BCG-vaccinated group (group C) alone. There were no associations between BCG recovered from biopsies by qPCR and levels of IgM (data not shown).

### Distinct Transcriptomic Profiles Are Associated With Good vs. Poor Mycobacterial Control in the MGIA

**Figure 6:**
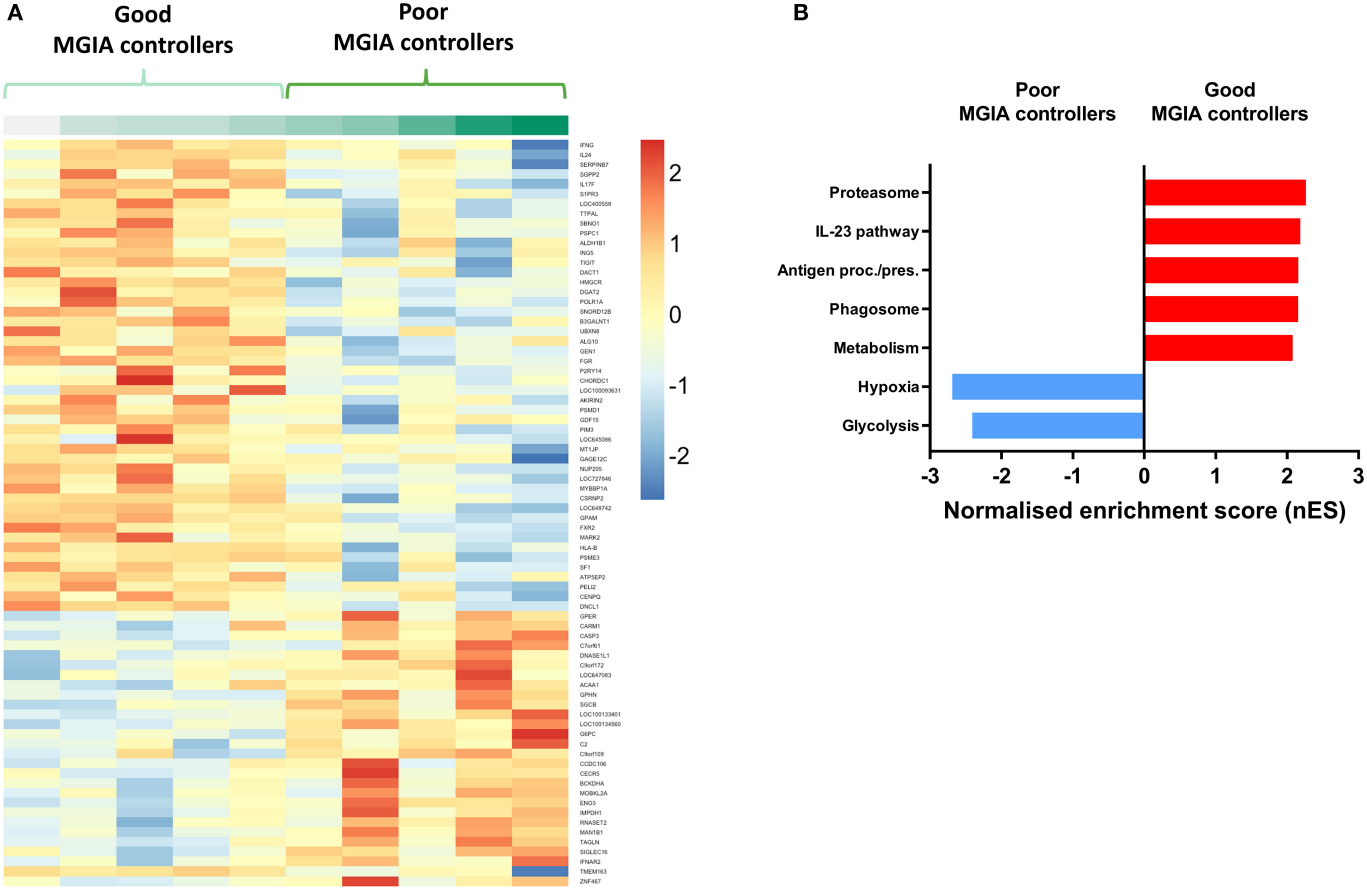
Distinct transcriptomic profiles are associated with good vs. poor mycobacterial control in the MGIA. Samples were taken from 10 volunteers assigned to group C (historically BCG vaccinated) in Study 2. Volunteers were infected with intradermal BCG and gene expression microarray analysis was conducted on PBMC taken at 2 weeks post-infection. The direct PBMC MGIA was conducted on cells and plasma taken at the day of challenge and the ratio of mycobacterial growth at the end of the 96 h culture relative to an inoculum control was determined. Volunteers were classified as ‘good' or “poor” controllers defined as having MGIA mycobacterial growth values below or above the group median, respectively. Gene expression was analyzed using a 2 × 2 factorial design with the interaction term (BCG stimulated − unstimulated PBMC for good MGIA controllers) vs. (BCG stimulated − unstimulated PBMC for poor MGIA controllers). Differentially expressed genes were defined as those with a *p* < 0.01 and log_2_ FC > 0.5 and are presented as a heatmap **(A)**. GSEA analysis was conducted to identify biological pathways that were enriched in “good” vs. “poor” controllers and vice versa using an FDR cut-off of < 0.05. Representative top GSEA pathways for each category are shown from the top 50 gene sets ranked by normalized enrichment score (nES) **(B)**.

Volunteers from the BCG-vaccinated group (Group C) from Study 2 (shown in Figure 2A) were classified dichotomously as “good” or “poor” controllers defined as having MGIA mycobacterial growth ratio values below or above the group median, respectively at day of infection. Two volunteers were excluded due to missing transcriptomic data for either the unstimulated or BCG-stimulated conditions. Control of mycobacterial growth was significantly different between the two sub-groups (*p* = 0.005, unpaired *t*-test) with no overlap in 95% confidence intervals (CI) (95% CI = [0.89, 0.92] and [0.93, 1.04] for “good” and “poor” controllers, respectively); data not shown. Time since BCG vaccination and demographic parameters including age, sex and ethnicity did not differ between sub-groups (Table 1). Transcriptomic analysis was conducted on PBMC taken at 2 weeks post-infection. Gene expression was analyzed using a 2 x 2 factorial design with the interaction term (BCG stimulated − unstimulated PBMC for good MGIA controllers) vs. (BCG stimulated − unstimulated PBMC for poor MGIA controllers). Eighty-two differentially expressed genes were identified (*p* < 0.01 and log FC > 0.5); after removing unannotated and duplicated genes, 76 remained: 49 of which were upregulated in good vs. poor controllers, and 27 of which were downregulated (Figure 6A). Top genes upregulated in good vs. poor controllers included IFN-γ, IL-17F, and IL-24, while those downregulated in good vs. poor controllers included IFNAR2 and Transgelin (TAGLN). The full list of significantly differentially expressed genes is provided in Supplementary Table 1.

**Table 1:** Demographic data for “good” vs. “poor” controllers taken from group C (historically BCG vaccinated) from Study 2 used in the transcriptomic analysis.

	**“Good” controllers (*n* = 5)**	**“Poor” controllers (*n* = 5)**
Age, years	23 (6.80)	23 (8.11)
Time since BCG vaccination, years	11 (10.42)	10 (10.21)
Sex, male	3 (60%)	2 (40%)
Ethnicity, white British	5 (100%)	5 (100%)

*Data are median (SD) or n (%)*.

Secondary gene-set enrichment analysis (GSEA) using the C2 curated gene sets and an FDR cut-off of < 0.05, identified pathways that were unique to each sub-group. The top 50 pathways were ranked by normalized enrichment score and categorized according to function and/or overlap in constituent genes. Good controllers showed enrichment of 135 gene sets compared with poor controllers; top gene sets included the IL-23 pathway and antigen processing/presentation (Figure 6B). Poor controllers showed enrichment of 21 gene sets compared with good controllers which were almost exclusively associated with hypoxia and glycolysis metabolism (Figure 6B). The full lists of significantly positively and negatively enriched gene sets are provided in Supplementary Tables 2, 3 respectively.

## Discussion

We have demonstrated a correlation between mycobacterial growth inhibition in the direct PBMC MGIA and protection from *in vivo* BCG infection in humans across two independent studies. On a group level, MGIA responses mirrored BCG recovery from biopsy, with BCG-vaccinated volunteers demonstrating significantly improved protection compared with naïve or MVA85A-vaccinated volunteers. The BCG-induced MGIA effect was stronger in Study 1, which may reflect the shorter time since immunization in the historically BCG-vaccinated volunteers in this study (median of 4 years) compared with those in Study 2 (median of 10 years). However, in Study 2, volunteers who were historically vaccinated with BCG and boosted with MVA85A showed significantly lower BCG recovery from biopsy compared with those who were vaccinated with MVA85A alone, which was not reflected in the MGIA. This may indicate lower sensitivity of the MGIA, as may be expected of an *in vitro* assay that can only model *in vivo* complexity to a certain extent. Importantly, we also observed a correlation between mycobacterial growth in the MGIA and BCG recovery from biopsy by qPCR or culture CFU at the level of the individual BCG-vaccinated volunteers. This data contributes toward the biological validation of the direct PBMC MGIA as a correlate of protection, and to our knowledge is the first report of any MGIA correlating with *in vivo* protection on a per-individual basis. It is also the first time an MGIA has been evaluated in relation to *in vivo* mycobacterial growth using a controlled human mycobacterial infection.

Although murine studies have previously shown an association between mycobacterial control in the MGIA and protection from *in vivo M.tb* challenge, it was on a per-group basis, replicating the pattern observed between experimental groups (25–27). It is not possible to correlate MGIA outcome and protection on an individual basis in mice or other small animal models, as animals must be sacrificed to provide bone marrow macrophages and/or splenocytes for the MGIA. However, in humans, post-vaccination blood samples can be obtained on the day of mycobacterial infection, providing matched MGIA and protection data. Given the variable efficacy of BCG, it is both more accurate and more stringent to correlate with *in vivo* protection on a per-individual basis. Humans also represent the target species for a TB vaccine candidate. As it is unclear whether outcomes of preclinical animal models for TB are predictive of those in humans (28), it is most desirable to develop a correlate of protection that can be applied to human clinical trials as well as preclinical studies and bridge between the two. The direct MGIA has demonstrated cross-species potential to maximize utility and comparability (9). One limitation of our studies is the use of BCG as a surrogate for virulent *M.tb* in the MGIA co-culture. However, MGIA outcomes using BCG and *M.tb* have previously been shown to strongly correlate (9, 29, 30), and BCG circumvents the need for biosafety level 3 (BSL3) facilities thus increasing transferability potential of the assay.

As we were interested in assessing the MGIA as an *ex vivo* model of infection, our analysis of associations between the MGIA response and immune parameters focussed on those measured at 2 weeks post-infection. Volunteers in this study were historically BCG-vaccinated and therefore we did not have the opportunity to measure the immune response to their initial vaccination. It is possible that immune measures in the weeks and months following initial vaccination would predict MGIA and/or *in vivo* infection outcome; though not possible to address here, further studies are planned in this regard. In addition to combining experimental groups, we also measured correlations with MGIA in our group of interest alone: BCG-vaccinated volunteers (group C), to ensure that associations were not artifacts of broad group differences. This group was selected as BCG is known to be efficacious in the UK population under study (4), and these volunteers showed a signal of protection in our *in vivo* and *in vitro* infection studies.

Furthermore, 2 weeks is an early time-point to detect an effector response, as indicated by the very low or undetectable frequencies of cytokine-producing T cells or specific antibodies observed in the naïve group, hence group A was not appropriate for exploring correlates of adaptive immunity. Unfortunately 2 weeks post-infection was the latest time of sampling, and therefore it was too early to observe measurable adaptive responses to controlled BCG infection in naïve volunteers. One might hypothesize that at a later post-infection time-point, these volunteers would show increased effector and memory T cell responses but that these may remain inferior to groups C and D who essentially received a BCG ‘boost', and that control in the MGIA may correlate at an individual level within these groups as currently observed in group C. The group vaccinated with MVA85A alone (group B) had low levels of protection from BCG infection in this study (20), and group D (historical BCG vaccination followed by MVA85A boost) did not show improved protection over group C (BCG vaccination alone); a result consistent with the findings of the infant efficacy trial (7). While group C and group D did not differ in CFU recovered from biopsy or MGIA responses, group D showed significantly lower ICS, ELISpot and ELISA responses than group C at 2 weeks post-infection. However, fold change in these measures between baseline and post-infection did not differ between these groups, suggesting some baseline differences between the small number of volunteers randomized to each group.

We found that the magnitude of the PPD-specific IFN-γ ELISpot response was inversely associated with mycobacterial growth in the MGIA in BCG-vaccinated volunteers. A similar association was previously reported in the same study between the IFN-γ ELISpot response and CFU recovered from biopsy following controlled infection (20), suggesting consistency between parameters contributing to the *in vivo* and *in vitro* models. A central role for IFN-γ in the immune response to TB is well-established (31–34), and our findings suggest that it may contribute to bacterial clearance from the site of *in vivo* infection and control of mycobacterial growth following *in vitro* infection, although it is yet to be determined which cell populations are contributing to IFN-γ secretion, and cell phenotyping would be of interest. Previous studies have not identified an association between specific ELISpot responses and the outcomes of various MGIAs, including the direct PBMC MGIA (10, 12, 14, 35, 36). This disparity is likely because those studies compared responses in samples taken at the same time-point, while we focussed on associations with post-infection responses. Measuring immune parameters post-infection permits consideration of re-stimulated memory responses in historically vaccinated volunteers. Since the MGIA models an infection, it follows that immune parameters induced by *in vivo* stimulation and contributing to control of mycobacterial replication at the site of BCG administration may reveal those driving control of mycobacterial growth in the MGIA.

We also identified associations between frequencies of total CD4+ T cells producing IFN-γ or TNF-α measured using ICS and improved control of mycobacterial growth in the MGIA in BCG-vaccinated volunteers. As previously reported, the frequency of CD4+ T cells producing IFN-γ or TNF-α also correlated significantly and negatively with BCG recovered by qPCR (23), suggesting a shared mechanism of immunity between the *in vivo* and *in vitro* models. Significant inverse associations were also observed between IFN-γ producing CD8+ T cells and BCG recovered from biopsy (23) or growth in the MGIA, suggesting that CD8+ T cells also represent a source of IFN-γ contributing to protection.

Although IFN-γ is known to be necessary for protection against TB, it may not be sufficient or reliable as a correlate of protection (31–34, 37, 38). We therefore considered PPD-specific polyfunctional CD4+ T cells, and identified IFN-γ, TNF-α, and IL-2 triple-positive cells as the dominant population at 2 weeks post-BCG infection. This is consistent with the findings of Smith *et al*., who observed these cells to be most abundant in BCG-vaccinated UK infants at 4 months and 1 year post-vaccination (13), although others have reported a more heterogeneous profile (39). This may be due to the study of different populations or the use of antigens other the PPD for stimulation. We saw significant inverse correlations between mycobacterial growth in the MGIA and the frequencies of several subsets of polyfunctional CD4+ T cells including IFN-γ, TNF-α, and IL-2 triple-positive cells, which is also consistent with Smith et al. (13). Notably, the strongest association was with IFN-γ and TNF-α double-cytokine producing cells which represent an effector memory population (40), supporting our hypothesis that parameters measured post-infection correlate with MGIA responses because both are measuring re-stimulated memory responses.

While others have reported no correlation between MGIA response and polyfunctional T cells (14, 26), we suggest that this may be due to the measurement of effector responses soon after vaccination, while we also captured vaccine-induced memory responses re-stimulated by *in vivo* infection. There is currently no consensus on whether polyfunctional T cells represent a marker of protective immunity or TB disease activity (41), but our data indicate that they may contribute to control of *in vivo* and *in vitro* infection with BCG. A limitation of these studies is that PPD was used as the stimulant in the ICS studies, while BCG was used for the *in vivo* and *in vitro* infections. Although stimulation with BCG in the whole blood ICS may facilitate more direct comparison with the MGIA, we (and others) have observed similar and corresponding responses to stimulation with PPD vs. BCG in gene expression profiles, ELISpot, ICS and ELISA responses (42–45). Furthermore, ICS was conducted using whole blood, while the MGIA was conducted in the PBMC compartment. However, we observed very low responses using cryopreserved PBMC ICS, which is known to be less sensitive (43, 46, 47), and were unable to detect any associations.

The levels of BCG-specific IgG, and to a lesser extent IgA, antibodies induced by BCG infection were significantly higher in the BCG-vaccinated group compared with naïve volunteers or those receiving MVA85A vaccination. This is consistent with several previous studies reporting the induction of specific antibodies following BCG vaccination [(48–51) and recently reviewed in (52)]. Levels of IgG following BCG infection were also weakly associated with improved control of mycobacterial growth in the MGIA in the BCG-vaccinated volunteers and with *in vivo* clearance of BCG from the site of infection. Although humoral immunity has long been neglected in the field due to the intracellular nature of mycobacteria, there is a growing body of evidence indicating a role for B cells and antibodies in immunity from TB ranging from modulation of the T cell response to antibody-dependent cellular phagocytosis and cytotoxicity (53, 54). O'Shea et al. reported a study of individuals with active TB disease or latent TB infection (LTBI) in which IgG1 responses to *M.tb-*specific antigens correlated with improved mycobacterial control in the direct whole blood MGIA (29). Our findings indicate that antibodies in the autologous serum or plasma added to the PBMC MGIA may contribute to the overall outcome.

Our transcriptomic analysis revealed distinct patterns of immune activation in response to specific antigen stimulation which were associated with differential ability to control mycobacterial growth in the MGIA in BCG-vaccinated volunteers. A number of genes of interest were identified as significantly upregulated following *in vivo* infection in “good” compared with “poor” MGIA controllers including those encoding the cytokines IFN-γ, IL-17F and IL-24 which are expressed by activated T cells and have been previously associated with control of TB infection (32, 33, 55, 56). Expression of these genes was also previously associated with BCG growth *in vivo* following BCG infection in these volunteers (23). Applying GSEA, one of the top gene sets enriched in good controllers was the IL-23 pathway, which has been implicated in the expression of the Th17 population and IL-17 response and control of mycobacterial infection (57–59). Others including antigen processing/presentation and the proteasome are consistent with our ELISpot and ICS findings indicating a central role for the cellular response in controlling mycobacterial growth.

Interestingly, gene sets found to be enriched in poor MGIA controllers were principally related to hypoxia/the hypoxia inducible factors (HIF) and associated metabolic reprogramming toward increased glycolysis. Several studies suggest a protective role for hypoxia during *M.tb* infection (60, 61). However, hypoxia is amplified as a result of inflammation, and a hypoxia-dominated expression profile may be indicative of ‘too much' inflammation, resulting in suppression of the beneficial T cell response and consistent with recent reports linking high levels of immune activation with risk of TB disease (62–64). Alternatively, hypoxia may be directly limiting the cellular response in these volunteers; indeed HIF-1α has been shown in some studies to inhibit the differentiation, proliferation and IFN-γ/IL-2 production of Th1 cells (65–67). The contrasting enrichment patterns may simply reflect qualitatively or temporally different responses to BCG infection, and it should be noted that the expression profiles of cells taken 2 weeks post-challenge are confounded by bacterial load in those individuals at that time, although it would be difficult to control for this biologically. While the contradictory literature on the effect of hypoxia complicates interpretation, the prominence of such pathways in our analysis supports further exploration of the role of hypoxia in TB infection and the potential utility of hypoxia-related host-directed therapies (61). A limitation of the transcriptomic data is that PBMC were stimulated with BCG rather than *M.tb*, although this may improve comparability with data from our *in vivo* and *in vitro* BCG infections, both of which used BCG.

The direct whole blood MGIA was previously performed on fresh samples from Study 2 but no differences between groups were identified and there was no association between mycobacterial growth and BCG recovery from biopsy by qPCR or CFU (20). The disparity between this and the current findings using PBMC may be due to greater inter-assay variability in the whole blood assay which must be run in real-time compared with the cryopreserved PBMC assay which was run in just two batches. Indeed, in a previous comparison of repeated baseline sampling using whole blood or PBMC, we saw improved inter-assay reproducibility in the PBMC assay (12). Furthermore, we have identified an influence of hemoglobin (Hb) and iron on the direct MGIA (68). While variation in clinical parameters including Hb levels forms part of the overall picture for clinical cohort studies and whole blood is applicable in this context (29, 69), it may confound measures of vaccine-induced control of mycobacterial growth and reduce sensitivity to detect a vaccine response (68).

In conclusion, we have demonstrated that the direct PBMC MGIA correlates with *in vivo* protection from controlled mycobacterial infection in humans on a per-individual as well as per-group basis. Furthermore, we have identified immune parameters post-*in vivo* BCG infection that are associated with improved control in the MGIA post-*in vitro* BCG infection. While it has been suggested that control of mycobacterial growth in the MGIA is driven by trained innate immunity (14), our cellular and transcriptomic data indicate that the adaptive immune response, particularly Th1 cells, also strongly influences outcome in BCG-vaccinated volunteers. The MGIA is a complex functional assay, likely driven by a combination of multiple interacting aspects of immunity, which is one of its advantages over measuring predefined individual parameters. Further validation studies are now required for both the controlled human infection model and the *in vitro* direct PBMC MGIA. In particular, it is important to evaluate the performance of the optimized models in trials of novel TB vaccine candidates and in different study populations. However, the data presented offer an indication that the direct PBMC MGIA is measuring a biologically relevant response and has the potential to represent a correlate of protection (or a tool to identify other relevant biomarkers) in the down-selection of vaccine candidates early in clinical development. Such a correlate could accelerate optimization and selection of vaccine candidates and may ultimately accelerate licensure of a new vaccine.

## Data Availability Statement

The transcriptomics datasets generated for this study can be found in the GSE58636.

## Ethics Statement

The studies involving human participants were reviewed and approved by the relevant ethics committees as follows: Study 1 was approved by Oxfordshire Research Ethics Committee A (REC reference 07/Q1604/3). Study 2 (NCT01194180) was approved by the Medicines and Healthcare Products Regulatory Agency (EudraCT 010-018425-19) and the Berkshire Research Ethics Committee (REC reference 10/H0505/31). The volunteers provided their written informed consent to participate in this study.

## Author Contributions

RT, HF, and HM contributed to the conceptualization and methodology. RT, IS, SH, MO'S, AC, MM, RW, AM, and JM performed investigative roles. RT, IS, DC, DO'C, MM, and HF performed formal analysis. HF and HM provided supervision. RT wrote the paper. All authors provided critical review and editing.

### Conflict of Interest

The authors declare that the research was conducted in the absence of any commercial or financial relationships that could be construed as a potential conflict of interest.

## Supplementary Material

Supplementary Figure 1 Gating strategy for ICS. Stimulated and unstimulated, fixed, lysed, and stained whole blood samples were acquired on the LSRII cytometer (BD) and data was analyzed using FlowJo version 8.8.7 (Tree Star Inc., Ashland, USA). Singlet, CD3+ and CD14– lymphocytes were chosen (panels 1–3). Panels 5–7 show IFN-γ and TNF-α responses in unstimulated, PPD-stimulated and SEB-stimulated CD4+ T cells, respectively. Panels 8–10 show IFN-γ and TNF-α responses in unstimulated, PPD-stimulated and SEB-stimulated CD8+ T cells respectively.

Supplementary Figure 2 Specific IFN-γ ELISpot responses are associated with improved control of mycobacterial growth in the MGIA. Samples were taken from Study 2. *n* = 48 healthy human volunteers were assigned to groups A and B (BCG-naïve) or groups C and D (historically BCG vaccinated). Groups B and D received the candidate TB vaccine MVA85A. All volunteers were then infected with intradermal BCG, and PPD-specific IFN-γ ELISpot responses were measured at 2 weeks post-infection **(A)**. The association between mycobacterial growth in the direct PBMC MGIA (conducted on cells and plasma taken at the day of challenge) and the PPD-specific IFN-γ ELISpot response was determined for all groups combined **(B)** and for the BCG-vaccinated group (group C) only **(C)**. Bars represent the median values with IQR. For **(A)** a one-way ANOVA with Tukey's multiple comparisons test was performed where ^**^*p* < 0.005, ^***^*p* < 0.0005, and ^****^*p* < 0.0001. For **(B,C)** a Spearman's correlation was performed. MGIA growth ratio = log_10_(CFU of sample/CFU of control).

Supplementary Table 1 List of significantly differentially expressed genes in good vs. poor MGIA controllers. Samples were taken from the BCG-vaccinated group (Group C) from Study 2. Full transcriptomic and MGIA data was available from *n* = 10 volunteers, which were classified dichotomously as “good” or “poor” controllers defined as having MGIA mycobacterial growth ratio values below or above the group median, respectively, at day of infection. Gene expression profiles of PBMC taken at 2 weeks post-infection were analyzed using a 2 × 2 factorial design with the interaction term (BCG stimulated − unstimulated PBMC for good MGIA controllers) vs. (BCG stimulated − unstimulated PBMC for poor MGIA controllers). Eighty-two differentially expressed genes were identified (*p* < 0.01 and log FC > 0.5) and are shown ranked by *p*-value.

Supplementary Table 2 List of significantly enriched gene sets in good vs. poor MGIA controllers. Gene-set enrichment analysis (GSEA) was performed using the C2 curated gene sets and an FDR cut-off of < 0.05. Good controllers showed enrichment of 135 gene sets compared with poor controllers, which are shown here ranked by normalized enrichment score (nES). The top 50 pathways were categorized according to function and/or overlap in constituent genes. Pathways shaded in gray could not be meaningfully categorized in this context.

Supplementary Table 3 List of significantly enriched gene sets in poor vs. good MGIA controllers. Gene-set enrichment analysis (GSEA) was performed using the C2 curated gene sets and an FDR cut-off of < 0.05. Poor controllers showed enrichment of 21 gene sets compared with good controllers, which are shown here ranked by normalized enrichment score (nES). Pathways were categorized according to function and/or overlap in constituent genes; those shaded in gray could not be meaningfully categorized in this context.
