# Supplementary figures and images for: Tools for Assessing the Protective Efficacy of TB Vaccines in Humans: in vitro Mycobacterial Growth Inhibition Predicts Outcome of in vivo Mycobacterial Infection
Source: Front Immunol. 2020 Jan 10;10:2983. doi: 10.3389/fimmu.2019.02983 (PMC6968127; doi:10.3389/fimmu.2019.02983)

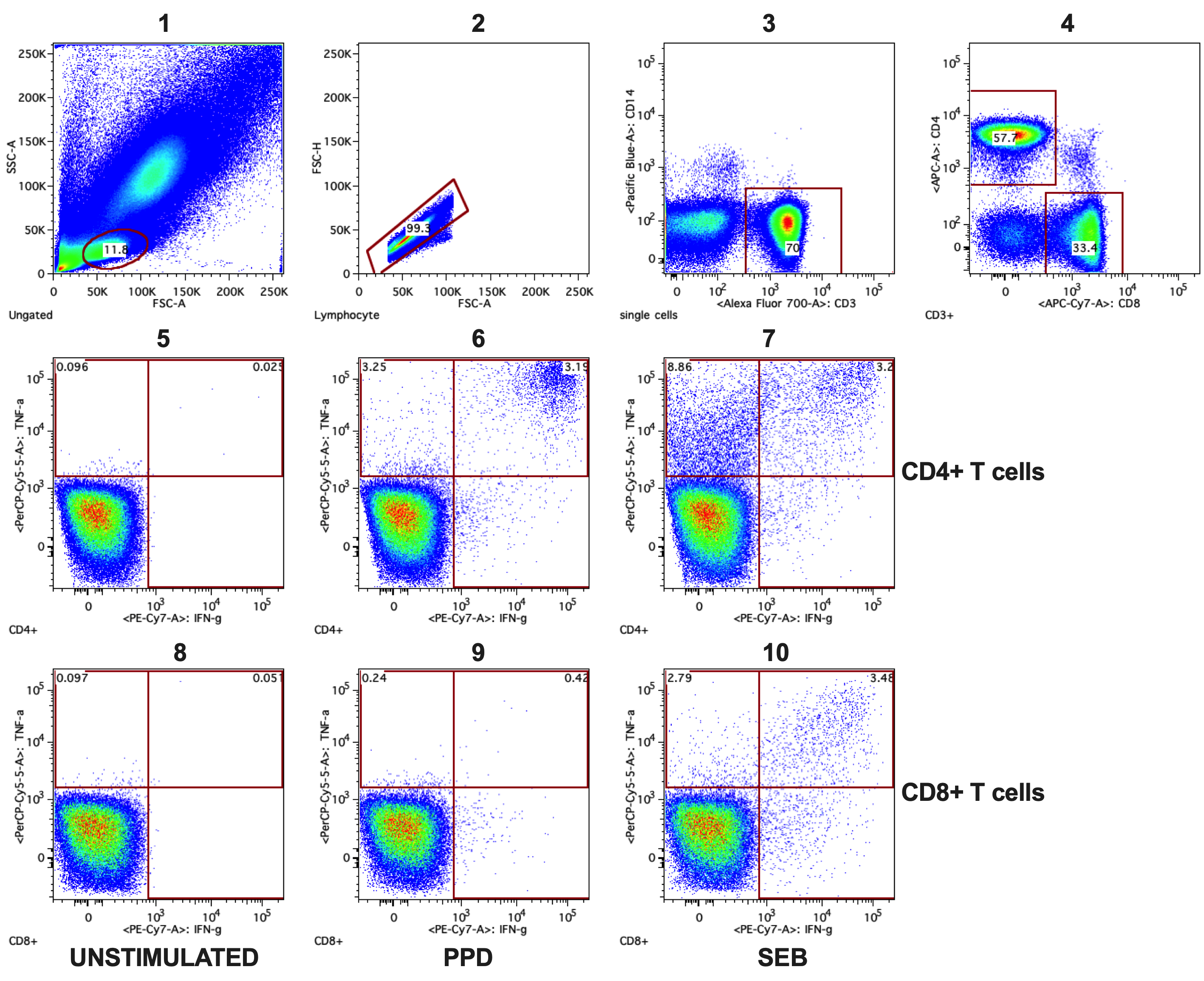

Supplement: Supplementary Figure 1 — Gating strategy for ICS. Stimulated and unstimulated, fixed, lysed, and stained whole blood samples were acquired on the LSRII cytometer (BD) and data was analyzed using FlowJo version 8.8.7 (Tree Star Inc., Ashland, USA). Singlet, CD3+ and CD14– lymphocytes were chosen (panels 1–3). Panels 5–7 show IFN-γ and TNF-α responses in unstimulated, PPD-stimulated and SEB-stimulated CD4+ T cells, respectively. Panels 8–10 show IFN-γ and TNF-α responses in unstimulated, PPD-stimulated and SEB-stimulated CD8+ T cells respectively. [file Image_1.TIFF]

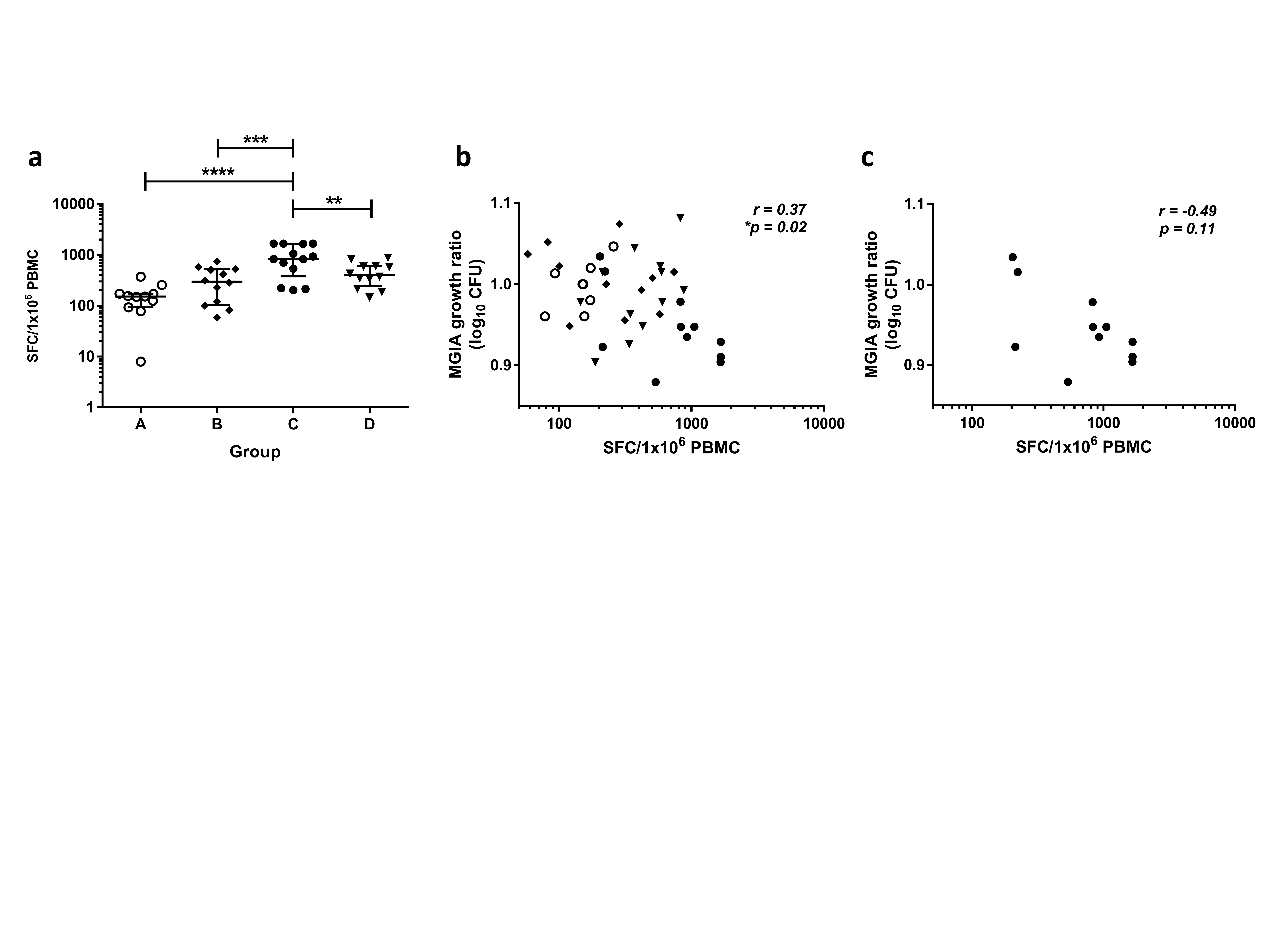

Supplement: Supplementary Figure 2 — Specific IFN-γ ELISpot responses are associated with improved control of mycobacterial growth in the MGIA. Samples were taken from Study 2. n = 48 healthy human volunteers were assigned to groups A and B (BCG-naïve) or groups C and D (historically BCG vaccinated). Groups B and D received the candidate TB vaccine MVA85A. All volunteers were then infected with intradermal BCG, and PPD-specific IFN-γ ELISpot responses were measured at 2 weeks post-infection (A). The association between mycobacterial growth in the direct PBMC MGIA (conducted on cells and plasma taken at the day of challenge) and the PPD-specific IFN-γ ELISpot response was determined for all groups combined (B) and for the BCG-vaccinated group (group C) only (C). Bars represent the median values with IQR. For (A) a one-way ANOVA with Tukey's multiple comparisons test was performed where **p < 0.005, ***p < 0.0005, and ****p < 0.0001. For (B,C) a Spearman's correlation was performed. MGIA growth ratio = log10(CFU of sample/CFU of control). [file Image_2.TIF]
